# Do Program Directors of Anesthesiology Residency Programs Interpret Narrative Letters of Recommendation as Intended?

**DOI:** 10.7759/cureus.63573

**Published:** 2024-07-01

**Authors:** Samantha R Pianello, Joanna M Abouezzi, Garret M Weber, Elizabeth Drugge, Marvin S Medow, Apolonia E Abramowicz

**Affiliations:** 1 Anesthesiology, New York Medical College, Valhalla, USA; 2 Anesthesiology, Westchester Medical Center, Valhalla, USA; 3 Public Health, New York Medical College School of Health Sciences and Practice, Valhalla, USA; 4 Pediatrics/Physiology, New York Medical College, Valhalla, USA

**Keywords:** survey, electronic residency application service, residency application, recommendation letters, residency, medical education, anesthesiology

## Abstract

Background

Letters of recommendation (LORs) are an important part of the application process for medical residency programs with most specialties preferring a narrative format. Given the inherent subjectivity of narrative LORs, the current study sought to determine whether the intended messages of narrative LORs written for applicants to anesthesiology residency programs are accurately interpreted by readers.

Methodology

Anonymous online surveys were sent via the Qualtrics platform to program directors (PDs) of the Accreditation Council for Graduate Medical Education-accredited anesthesiology residency programs in the Mid-Atlantic region as designated by the Electronic Residency Application Service, which consists of the states of New York, Pennsylvania, and New Jersey. Each PD participant received five surveys, each of which was attached to a de-identified LOR that was written by another PD located at an institution in the same region. Both the letter writer and study participants were asked to score LORs on a Likert-like scale. Participants were additionally asked whether the LORs, if received, would influence their decision to either offer an interview to the applicant or to rank the applicant. Finally, participants were asked to note any specific words or phrases within the LORs that they found to be particularly impactful.

Results

Overall, 10 of 34, 29.41%, PDs responded to the survey. There was a high correlation between the LOR intent and the respondents’ assigned rating (Spearman’s rho = 0.7973, p < 0.001). Responses were more accurate for “outstanding and excellent” LORs compared to the lower three categories. Results were unaffected after adjusting for respondents’ years of experience as PDs. Additionally, 71.6% indicated that the LORs would influence the decision about offering an interview, and 56.5% stated that the LORs would influence a ranking decision.

Conclusions

Our results indicate that respondents’ perception of LORs correlated strongly with the intent of the writer. Additionally, respondents seemed to value LORs for interview and ranking decisions.

## Introduction

Letters of recommendation (LORs) have traditionally been considered a crucial factor in the selection of medical students applying for residency. In surveys of residency program directors (PDs) and selection committees across specialties, LORs are often cited as being one of the most important parts of a candidate’s application [1]. This holds true in anesthesiology, in which the 2021 National Resident Matching Program (NRMP) PD Survey found that PDs consider LORs in the specialty to be of high importance, with 78.6% citing them as a factor in determining which candidates to interview [1]. Narrative LORs are the preferred format for most specialties, including anesthesiology, although they are limited by inconsistent and potentially “coded” language, writer bias toward the applicant, and overwhelmingly positive statements that make it difficult to distinguish between applicants who are and are not competitive for programs [2-6]. In recent years, efforts have been made to find more objective ways to assess LORs, most notably via the introduction of standardized LORs used in specialties such as otolaryngology and emergency medicine [6-8]. However, the utility of depending on standardized letters has been limited by score inflation and lack of adherence to compositional guidelines [8-10]. The need for more objective evaluations in resident selection in the coming years is heightened by the recent transition from a scored United States Medical Licensing Exam (USMLE) Step 1 to a pass/fail system in January 2022 [11,12].

In addition to the aforementioned limitations of narrative LORs, they are also subject to a high degree of interpretive variability. In assessing inter-reader reliability in the evaluation of narrative LORs, Dirschl and Adams (2000) found low to moderate agreement between readers [13]. When evaluating applications for orthopedic surgery programs, Egan et al. (2021) identified low correlations between writers’ intended messages and readers’ interpretations, as well as similarly low agreement between readers’ appreciation of letters [14]. The current study seeks to assess whether PDs in graduate medical education programs specifically within the field of anesthesiology accurately interpret LORs as intended by the writer using narrative letters.

## Materials and methods

This study was deemed Exempt according to 45CFR46.101(b) (2): Category 2 by the New York Medical College General Medical and Behavioral Institutional Review Board (approval number: 19512).

Our three main objectives in performing this study were to use a narrative type of letter to explore the (1) interpretation of LORs by PDs, (2) whether the LORs, if received, would influence PDs’ decision to offer an interview to the applicant, and (3) whether the LORs, if received, would influence the PDs’ decision to rank the applicant on the program’s rank order list.

We first created a survey using Qualtrics, a secure online survey platform that allows for the creation of surveys and data collection and storage. It is password-protected and encrypted and allows for anonymous data collection. The survey was distributed to PDs via email directly through Qualtrics. We were not aware of which specific PDs had or had not completed the survey at any time during the study, only which email addresses had successfully received the survey.

The study included only core PDs at accredited anesthesiology residency programs in the Mid-Atlantic region designated by the Electronic Residency Application Service (ERAS), which includes the states of New Jersey, New York, and Pennsylvania. We chose this relatively small sample of PDs to reduce confounding variables associated with geographic regions, including possible variations in values and program culture that would be more likely in a sample of PDs at programs spread out over a wider geographic region. Program accreditation was verified through the Accreditation Council for Graduate Medical Education (ACGME) and FREIDA Residency Program databases. Email addresses for PDs were obtained via program websites or program coordinators. A total of 34 PDs were sent the survey.

**Figure 1:**
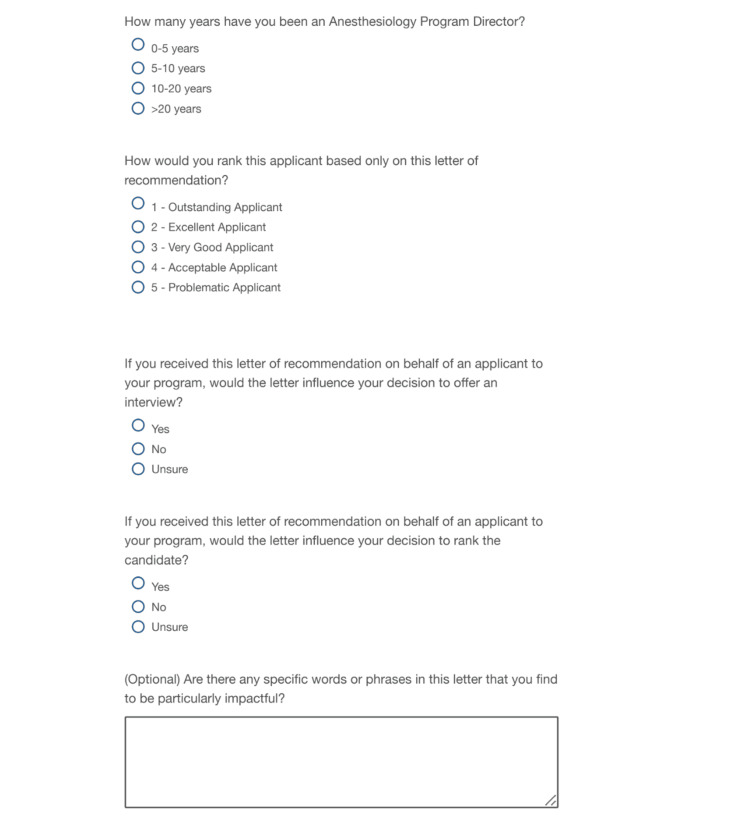
Qualtrics survey. The screenshot shows an example of the Qualtrics survey as it appeared to recipient program directors (PDs).

The survey was attached to each of the five LORs that were written for medical students who had applied to anesthesiology residency programs in previous years. To minimize stylistic variation, letters that were authored by a single PD were selected, and this PD chose five based on their intended difference in strength. The letters were then manually de-identified through the removal of all names, pronouns, institutions, and references to other demographic information such as race, ethnicity, and nationality, where applicable. Survey respondents were unaware of the fact that each letter was intended to correspond to a separate category. The survey required respondents to read each letter and score it on the following scale: 1 (outstanding applicant), 2 (excellent applicant), 3 (very good applicant), 4 (acceptable applicant), and 5 (problematic applicant). Participants were also asked if the letters, if received on behalf of applicants to their program, would influence their decisions to offer interviews or rank the candidates. The PD author used the same survey to assign intended scores to the letters. Additionally, the survey also asked how many years the respondent had been a PD. An optional question was also included, asking respondents to note specific words or phrases, if any, that stood out to them when reading each letter. The survey was limited to one submission per participant. Figure 1 shows the survey as it appeared to PDs who received it.

The duration of data collection was approximately 90 days. Periodic reminder emails were sent to participants to complete the study at two-week intervals. Completion of the survey was voluntary and anonymous, which was conveyed to participants via an introductory email that included a link to the survey. Participants were not able to proceed to the survey without consenting to participate. There was no compensation for participation in the study. Collected data were stored in Qualtrics, which is password-protected.

The study includes a Likert-type five-point scale, with categories of intended problematic, acceptable, very good, excellent, and outstanding applicant strength. Frequencies and percentages are reported for categorical variables. Spearman’s correlation analysis was used to assess the relationship between respondent rating and LOR intent. Pearson’s chi-squared test of independence was used to determine the effect of years of experience as a PD on PD rating. Mixed effects logistic regression was used to model “correct rating by PD” as a binary variable with LOR intention (Likert scale) and years of experience as a PD (ordinal categorical) as predictor variables and a random intercept by PD. Average predicted probabilities and 95% confidence intervals are reported. A p-value ≤0.05 was considered statistically significant. Data were collected in Excel and analyzed using STATA version 18 (StataCorp, College Station, TX, USA). Answers to the final qualitative survey question (“Are there any specific words or phrases in this letter that you find to be particularly impactful?”) were reported for each category.

## Results

Overall response rate

The survey was distributed to 34 anesthesiology residency PDs in the ERAS Mid-Atlantic region (New York, New Jersey, and Pennsylvania). There is evidence that applicants usually match to programs within their geographic region. The likelihood of responding PDs’ familiarity with the style of the letter author could have been enhanced by the focused distribution of the survey [15]. Of these 34 PDs, 10 responded to the survey anonymously, and one survey was partially completed. The overall response rate was 29.4%. Overall, 60% of respondents had fewer than 10 years of experience as an anesthesiology PD. The distribution of years of experience as PD is shown in Table 1.

**Table 1:** Years of experience of program director respondents. The Qualtrics survey included a question that asked respondents to note the range of years most accurately reflecting the length of time they have served as anesthesiology program directors. The table presents the results of this question; 60% of respondents (n = 10) had fewer than 10 years of experience.

Years of experience	%	N
0–5 years	30%	3
5–10 years	30%	3
10–20 years	20%	2
>20 years	20%	2
Total	100%	10

Is the perception of letter readers consistent with the intent of letter writers?

**Figure 2:**
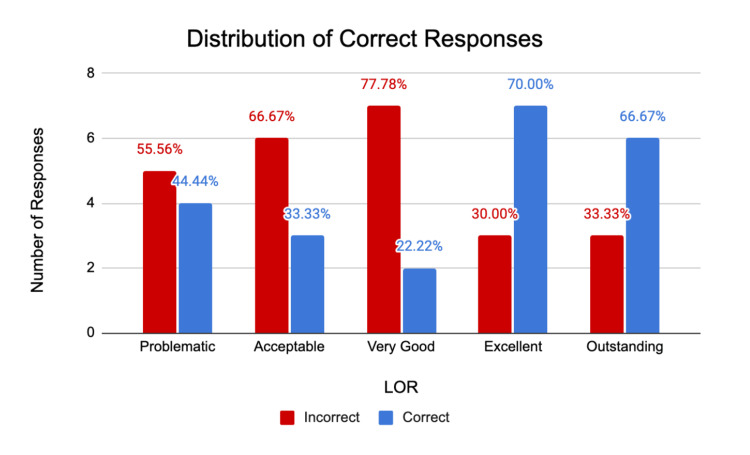
Distribution of correct versus incorrect responses for each letter of recommendation. Survey respondents were asked to score each of five letters of recommendation (LORs) on the following scale: problematic, acceptable, very good, excellent, and outstanding. These responses were then compared to those of the PD who authored the LORs. The distribution of “correct” versus “incorrect” responses is shown in the bar graph.

The survey asked participants to rate the included five letters on a scale of 1 through 5, indicating a rating of the applicant of 1 (outstanding applicant), 2 (excellent applicant), 3 (very good applicant), 4 (acceptable applicant), and 5 (problematic). When reading the letter for the intended “outstanding” applicant, six out of nine respondents correctly chose “outstanding” as the intended type of applicant. Two answered “excellent,” and one answered “very good.” When asked to rate the “excellent” applicant, seven out of 10 correctly identified the letter as “excellent,” two answered “very good,” and one answered “outstanding.” In scoring the “very good” letter, only two out of nine correctly matched the intended meaning, while six labeled the letter as “excellent” and one labeled it as “outstanding.” The “acceptable” applicant letter was rated equally “problematic,” “acceptable,” and “very good” (three out of nine in each category). Similarly, when rating the “problematic” letter, four participants labeled the letter as “problematic,” two as “acceptable,” and three as “very good.” Overall, 48% of letters were accurately categorized by respondents. Figure 2 shows the number of correct and incorrect responses for each of the LORs.

As shown in Figure 2, the “exceptional” and “outstanding” LORs were more likely to be correctly identified by readers compared to the LORs in the lower categories, with approximately 70% of respondents correctly rating the former. Additionally, even incorrect responses resulted in some stratification of the LORs; for example, the “outstanding” letter was rated by multiple readers as either “excellent” or “very good,” while incorrect ratings of the “problematic” letter were either “acceptable” or “very good.” LORs in the lowest two categories were never given the highest two ratings even when incorrectly rated. Additionally, LORs in the highest two categories were never given the lowest two ratings when incorrectly rated.

**Figure 3:**
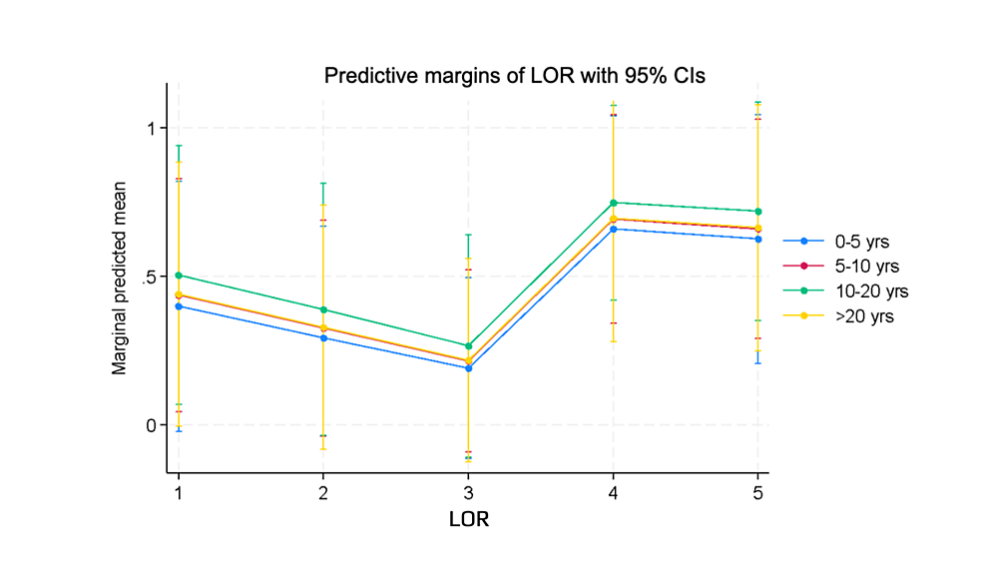
Marginal predicted mean of letters of recommendation adjusted for years as a program director. We used marginal predicted means to adjust our results shown in Figure 2 for years that respondents had served as anesthesiology program directors. We found no significant difference in the “correctness” of letter of recommendation ratings when adjusted for years of experience. LOR = letter of recommendation; CIs = confidence intervals

When we adjusted the correctness of responses for years respondents served as PD, the results showed no significant change in predictive success. These results are shown in Figure 3.

**Figure 4:**
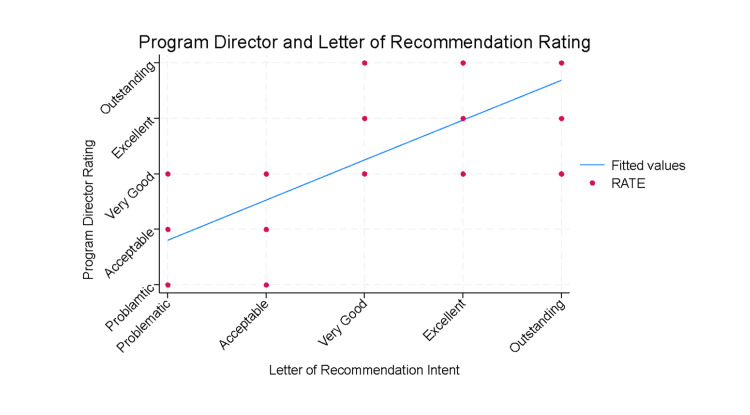
Pairwise correlation of letter of recommendation author’s intention and program director respondent’s rating. We used Spearman correlation analysis to assess the relationship between the letter of recommendation author’s intent and the program director rating of each of the five letters. We found a strong positive correlation between the two variables (Spearman correlation coefficient = 0.7973; n = 46, p < 0.001). The results are plotted in the graph.

Spearman’s correlation analysis, shown in Figure 4, revealed a relatively strong positive relationship between the LOR intent and the PD rating of each letter (ρ = 0.797, p < 0.001). This indicates a strong correlation between the intent and perception of the intent of the letters when evaluated by the responding PDs.

## Discussion

LORS are historically an important component of the residency application process, and their utility is likely increasing during the period of transition to the pass/fail scoring of USMLE Step 1. Despite their importance, LORs are frequently criticized as inherently biased, subjective, and containing coded language that can be difficult for readers to decipher. Prior publications have characterized some of this coded language, identifying particular words and phrases that stand out to anesthesiology letter readers [2,3]. The current study sought to further explore the messaging of LORs to determine whether the intent of anesthesiology LOR writers is accurately perceived/decoded by readers.

Writer versus reader agreement

Our results indicate a strong correlation between writer intent and reader perception, with a Spearman’s rho of 0.7973 (n = 46, p < 0.001). The correlation was higher for the “outstanding” and “excellent” LORs compared to the LORs in the lower three categories. This result may be artificially inflated by the fact that survey respondents were not instructed to assign a different rank to each letter, and several gave the same rating to multiple letters; however, in general, the intended messages of the writer of the LORs used in this study tended to be perceived accurately by the letter readers, particularly for highly rated LORs.

Role of LORs in applications

A majority of responses indicated that the letters used in the survey could influence the reader to offer an interview (71.4%), and the applicant’s position on the NRMP Rank Order List (56.5%). However, there were no notable patterns in the responses to the qualitative question regarding specific words and phrases that readers may have found to be impactful, other than the word “outstanding” in the letter that was assigned to the “outstanding” category by the writer. Multiple respondents commented on the overall tone of letters, or the absence of strong adjectives rather than the presence of particular words and phrases. These results underscore the inherent subjectivity of narrative LORs and the need for further study of letter structure and content to best convey candidate qualifications.

Further study

Given the small sample size of the study possibly stemming from the timing of the survey, future research on the topic may benefit from an extended period of data collection that primarily falls outside of the ERAS application season. PDs may be more likely to respond when they are not exceptionally busy preparing for the application cycle and reviewing applications. An additional benefit of this strategy could be to limit possible bias that might result from PDs responding to the survey while concurrently reading thousands of LORs for applicants to their own programs.

Other features of our study, such as its exclusive use of LORs that were written by a single writer and our decision to send the survey only to PDs in the ERAS Mid-Atlantic region, may benefit from expansion in future research to increase generalizability. We structured the study in this way to control for confounding variables such as writer background, experience, and values, as well as variations in vocabulary and tone, all of which may have influenced LOR reader perception and reduced survey reliability. We also de-identified applicant pronouns, cultural identifiers where applicable, and institutions within the body of the LORs to control for possible reader biases related to gender or other demographics. However, it should be noted that real-world application reviewers are necessarily reading LORs that were written by a large number of different people and include the background characteristics of the applicants. Thus, the generalizability of our study may be limited. It might be beneficial to use LORs from several writers, and even to explore the impact of the aforementioned variables on the results. Additionally, expanding this project to other regions or specialties, such as general surgery, could elucidate more valuable information regarding the impact of LORs on residency applications.

Finally, a LOR is only one part of a residency application, and applicants cannot necessarily be separated into distinct categories based on a single LOR. Nonetheless, LORs have been and will continue to play a larger role in the residency application process as fewer objective measures and numerical data, such as USMLE Step 1 scores, are reported.

Limitations

The greatest limitation of this study is its small sample size and possible nonresponse bias. As reported in the Results section, 29.4% of survey recipients responded. We speculate that the timing of survey distribution may have had an impact on our response rate; responses were recorded over a 90-day period from July to October, a time during which anesthesiology residency programs are typically preparing for and beginning the ERAS application cycle. Still, the response rate is comparable to the response rates of similar survey-based studies, including a recent study on narrative LORs (33.8%), as well as the 2022 NRMP Program Director Survey (33.1%) [1,2].

## Conclusions

Our results indicate that anesthesiology PDs’ perception of LORs correlated strongly with the intent of the letter writer, and responses were similar regardless of respondents’ years of experience as a PD. Notably, respondents were more accurate in rating “outstanding” and “excellent” letters in comparison to “problematic” and “acceptable” letters. Additionally, a majority of respondents valued LORs both for offering interviews and ranking applicants. However, there were no consistent patterns in respondents’ answers regarding particular words or phrases that they found to be impactful, with several respondents stating that overall tone or lack of strong descriptors were most notable for them as they read the letters, highlighting the high degree of subjectivity of LORs both on the end of the writer and the reader. Based on these results, which are limited by a small sample size, a follow-up expanded study to include more PDs over a wider geographic region, and perhaps even PDs from specialties outside of anesthesiology, could improve the generalizability of our results. Prior publications have demonstrated that there is a need for either agreed-upon wording or standardization of LORs, similar to the standardized letter of evaluation that is used in emergency medicine residency programs. Our findings further highlight a need for LOR standardization, particularly for average and lower-rated LORs, for which the correlation between intent and perception was not as strong as it was for more highly rated letters. Additional studies should be conducted to further characterize the ongoing role of LORs in the residency application process, which has a growing need for more objective measures of evaluation and stratification.

## References

[1] National Resident Matching Program, Data Release and Research Committee (2022). Results of the 2022 NRMP Program Director Survey. National Resident Matching Program. https://www.nrmp.org/wp-content/uploads/2022/09/PD-Survey-Report-2022_FINALrev.pdf.

[2] Jn Pierre CE, Weber GM, Abramowicz AE (2021). Attitudes towards and impact of letters of recommendation for anesthesiology residency applicants. Med Educ Online.

[3] Rajesh A, Rivera M, Asaad M (2019). What are we REALLY looking for in a letter of recommendation?. J Surg Educ.

[4] Saudek K, Saudek D, Treat R, Bartz P, Weigert R, Weisgerber M (2018). Dear Program Director: deciphering letters of recommendation. J Grad Med Educ.

[5] Grall KH, Hiller KM, Stoneking LR (2014). Analysis of the evaluative components on the Standard Letter of Recommendation (SLOR) in Emergency Medicine. West J Emerg Med.

[6] Love JN, Deiorio NM, Ronan-Bentle S, Howell JM, Doty CI, Lane DR, Hegarty C (2013). Characterization of the Council of Emergency Medicine Residency Directors' standardized letter of recommendation in 2011-2012. Acad Emerg Med.

[7] Perkins JN, Liang C, McFann K, Abaza MM, Streubel SO, Prager JD (2013). Standardized letter of recommendation for otolaryngology residency selection. Laryngoscope.

[8] Zywiel MG (2021). CORR Insights®: are narrative letters of recommendation for medical students interpreted as intended by orthopaedic surgery residency programs?. Clin Orthop Relat Res.

[9] Saudek K, Treat R, Goldblatt M, Saudek D, Toth H, Weisgerber M (2019). Pediatric, surgery, and internal medicine program director interpretations of letters of recommendation. Acad Med.

[10] Naples R, French JC, Lipman JM (2020). Best practices in letters of recommendation for general surgery residency: results of expert stakeholder focus groups. J Surg Educ.

[11] (2021). USMLE Step 1 transition to pass/fail only score reporting. https://www.usmle.org/usmle-step-1-transition-passfail-only-score-reporting.

[12] Whelan AJ (2020). The change to pass/fail scoring for Step 1 in the context of COVID-19: implications for the transition to residency process. Acad Med.

[13] Dirschl DR, Adams GL (2000). Reliability in evaluating letters of recommendation. Acad Med.

[14] Egan CR, Dashe J, Hussein AI, Tornetta P 3rd (2021). Are narrative letters of recommendation for medical students interpreted as intended by orthopaedic surgery residency programs?. Clin Orthop Relat Res.

[15] Love ER, Dexter F, Reminick JI, Sanford JA, Karan S (2021). Interview data highlight importance of "same-state" on anesthesiology residency match. Anesth Analg.

